# Integrated Analysis of Multiple Microarray Datasets Identifies a Reproducible Survival Predictor in Ovarian Cancer

**DOI:** 10.1371/journal.pone.0018202

**Published:** 2011-03-29

**Authors:** Panagiotis A. Konstantinopoulos, Stephen A. Cannistra, Helen Fountzilas, Aedin Culhane, Kamana Pillay, Bo Rueda, Daniel Cramer, Michael Seiden, Michael Birrer, George Coukos, Lin Zhang, John Quackenbush, Dimitrios Spentzos

**Affiliations:** 1 Division of Hematology/Oncology, Department of Medicine, Beth Israel Deaconess Medical Center, Harvard Medical School, Boston, Massachusetts, United States of America; 2 Department of Biostatistics and Computational Biology, Dana Farber Cancer Institute, Department of Biostatistics, Harvard School of Public Health, Boston, Massachusetts, United States of America; 3 Department of Obstetrics, Gynecology, and Reproductive Biology, Massachusetts General Hospital, Harvard Medical School, Boston, Massachusetts, United States of America; 4 Department of Obstetrics, Gynecology, and Reproductive Biology, Brigham and Women's Hospital, Harvard Medical School, Boston, Massachusetts, United States of America; 5 Fox Chase Cancer Center, Philadelphia, Pennsylvania, United States of America; 6 Division of Gynecologic Medical Oncology, Massachusetts General Hospital, Harvard Medical School, Boston, Massachusetts, United States of America; 7 Department of Gynecologic Oncology, University of Pennsylvania, Philadelphia, Pennsylvania, United States of America; 8 Department of Cancer Biology, Dana-Farber Cancer Institute, Boston, Massachusetts, United States of America; Baylor College of Medicine, United States of America

## Abstract

**Background:**

Public data integration may help overcome challenges in clinical implementation of microarray profiles. We integrated several ovarian cancer datasets to identify a reproducible predictor of survival.

**Methodology/Principal Findings:**

Four microarray datasets from different institutions comprising 265 advanced stage tumors were uniformly reprocessed into a single training dataset, also adjusting for inter-laboratory variation (“batch-effect”). Supervised principal component survival analysis was employed to identify prognostic models. Models were independently validated in a 61-patient cohort using a custom array genechip and a publicly available 229-array dataset. Molecular correspondence of high- and low-risk outcome groups between training and validation datasets was demonstrated using Subclass Mapping. Previously established molecular phenotypes in the 2^nd^ validation set were correlated with high and low-risk outcome groups. Functional representational and pathway analysis was used to explore gene networks associated with high and low risk phenotypes. A 19-gene model showed optimal performance in the training set (median OS 31 and 78 months, p<0.01), 1^st^ validation set (median OS 32 months versus not-yet-reached, p = 0.026) and 2^nd^ validation set (median OS 43 versus 61 months, p = 0.013) maintaining independent prognostic power in multivariate analysis. There was strong molecular correspondence of the respective high- and low-risk tumors between training and 1^st^ validation set. Low and high-risk tumors were enriched for favorable and unfavorable molecular subtypes and pathways, previously defined in the public 2^nd^ validation set.

**Conclusions/Significance:**

Integration of previously generated cancer microarray datasets may lead to robust and widely applicable survival predictors. These predictors are not simply a compilation of prognostic genes but appear to track true molecular phenotypes of good- and poor-outcome.

## Introduction

Epithelial ovarian cancer (EOC) presents an example of the promise and challenges of using microarray analysis for prognostic biomarker research. Based on its highly heterogeneous clinical course [1], [2], [3] (even within advanced EOC, which represents over 70% of cases) and the modest discriminatory power of conventional prognostic factors (amount of residual disease after initial surgery, age, tumor grade, and histologic subtype [1], [4], [5]), microarray studies were pursued in an attempt to account for the molecular and biologic complexity of the disease [6], [7], [8], [9], [10]. However, none produced a gene expression signature that has been appropriate for clinical use. This is largely due to, among other reasons, variable or small sample size, lack of adequate validation, or inclusion of subtypes (clear cell, mucinous, papillary EOCs), which constitute distinct molecular entities [11]. While collectively these studies may be sufficient to identify useful signatures, combining data or the analytical results is difficult for many reasons, including the use of a variety of array platforms, different data normalization and analysis approaches, and variability in experimental protocols and patient selection. Finally, in many instances it is not clear if the prognostic signatures reflect reproducible stable disease phenotypes or are simply a combination of prognostic genes. These limitations, which are not unique to ovarian cancer, demonstrate the challenges limiting the application of microarray signatures in cancer care and research, especially in cancers with more limited availability of appropriate tissue resources.

In an effort to address these challenges, we assembled, curated, and processed a collection of 265 raw gene expression arrays from four previously reported ovarian cancer microarray studies [10], [12], [13], [14] applying consistent data normalization, quality control, and analytical methods. A multi-gene model was identified in this composite set that was then independently validated in two separate tumor cohorts, one of which was profiled on a custom array genechip and the other was a publicly available standard oligonucleotide array dataset [15]. Finally, we showed that this multi-gene model is not simply prognostic of outcome but reflects reproducible ovarian cancer phenotypes and appears to simultaneously track deregulation of several biological or oncogenic pathways in this disease.

## Results

### Development of multi-gene prognostic classifiers in the integrated training set

**Figure 1:**
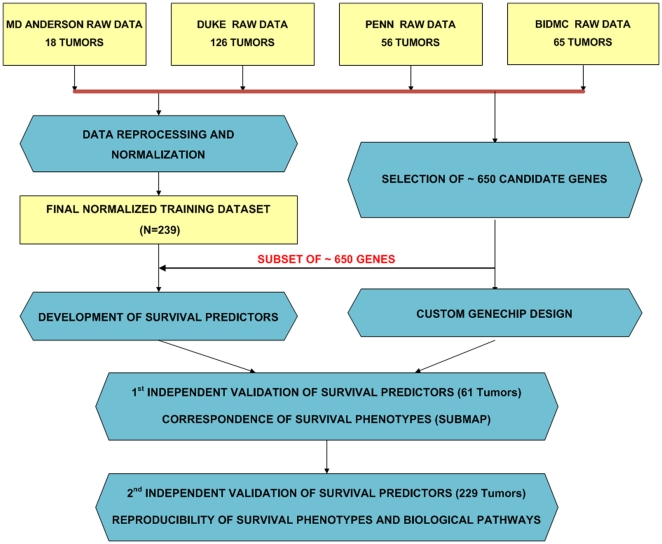
Consort Diagram (Study work flow). Raw data (Affymetrix .CEL files) from four previously reported microarray datasets from different institutions were used. Outlier samples were excluded and batch effect was adjusted resulting in the final training set (239 arrays). 650 genes were selected by performing survival analysis in each dataset and were used to develop prognostic models in the final training set. Data pre-processing (quality control and batch adjustment) and normalization resulting in an integrated training set was done separately from the selection of 650 genes, which were chosen independently by performing survival analysis in each of the 4 datasets (MD ANDERSON, PENN, DUKE, BIDMC). These preselected 650 genes were then used to develop prognostic models in the unified training set. These models were independently validated in two independent datasets: a 61-tumor cohort using a custom array containing the 650 preselected genes and a 229-tumor recently published ovarian cancer microarray dataset. The correspondence of the low- and high-risk phenotypes was assessed using SubMap.

**Figure 2:**
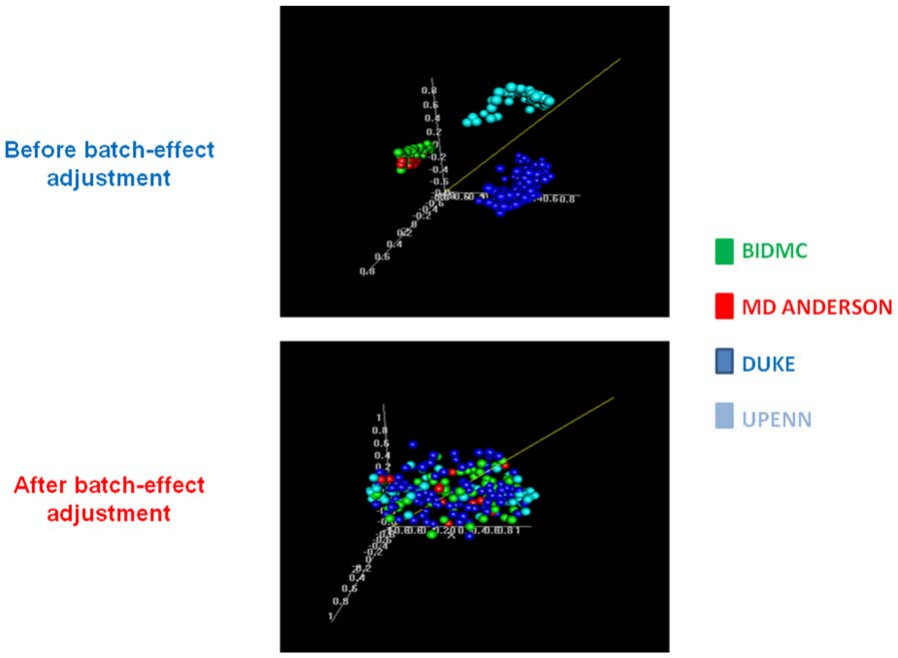
Adjustment for non-biological experimental variation. Multidimensional scaling of the combined training set revealed that, before application of the batch adjustment algorithm, each dataset clearly separated from all the others (“batch effect”), whereas after correction of batch effect, samples from all datasets were well intermixed.

Figure 1 shows the workflow of our study (consort diagram). We designed a custom array gene chip that included approximately 650 top performing candidate genes identified by applying the supervised principal component survival analysis in each of the four previously reported datasets. Then, we combined all four microarray datasets into a composite training set (excluding 39 outlier samples), which consisted of 239 tumor arrays (Table 1, Figure 1). Hierarchical clustering in the combined training set revealed that, before application of the batch adjustment algorithm, each dataset clearly separated from all the others reflecting non-biological experimental variation (“batch effect”), whereas after adjustment for batch effect, tumor samples from all datasets were well intermixed (Figure 2).

**Table 1:** Clinical and Pathological Characteristics of Training and Validation Cohorts.

Clinical and Pathological characteristics							
		INTEGRATEDTRAINING a		1st VALIDATION		2nd VALIDATION	
		Patients (n = 239)		Patients (n = 61)		Patients (n = 229)	
Characteristic		No.	%	No.	%	No.	%
**Age**	**Median**	58.5		63		59.3	
	**Range**	36–82		44–84		23–80	
**Grade**	**I**	9	4	0	0	0	0
	**II**	67	28	5	8	85	37
	**III**	144	60	56	92	144	63
**Debulking** **Status** b	**Optimal**	101	42	43	70	129	56
	**Suboptimal**	110	46	18	30	8	3
**Stage** c	**1**	0	0	0	0	12	5
	**2**	0	0	0	0	12	5
	**3**	204	85	47	77	191	83
	**4**	34	14	13	21	14	6
**Histology**	**Serous**	230	96	60	98	216	94
	**Endometrioid**	9	4	1	2	13	6
**Chemotherapy** d	**Platinum-Taxane**	175	73	61	100	171	75
	**Platinum-Cytoxan**	51	21	0	0	0	0
	**Platinum alone**	2	1	0	0	45	20
	**No chemotherapy**	0	0	0	0	13	6

a Data were not available for all patients in the integrated training set (grade was not available for 19 pts, debulking status for 18 pts, stage for 1 pt and chemotherapy for 11 pts).

b 8/229 (3%) of the tumors were definitely known to be suboptimally debulked in the 2^nd^ validation set. 129/229 (56%) were optimally debulked. 61/229 (27%) had <2 cm residual disease after surgery but is unclear how many had <1 cm, and for 31/229 (14%) debulking status was unknown.

c Stage was not available for one patient in the training and for 1 patient in the 1^st^ validation set.

d Chemotherapy information was not known in the training set for 11 patients.

We subsequently used the pool of the 650 marker genes (without knowledge of their performance on the custom array) in order to generate multi-gene prognostic classifiers in the combined training set. Genes associated with survival (p<0.05) were ranked based on their absolute Cox regression coefficients, and prognostic models with the top ranking genes were developed using supervised principal component survival analysis [16].

**Figure 3:**
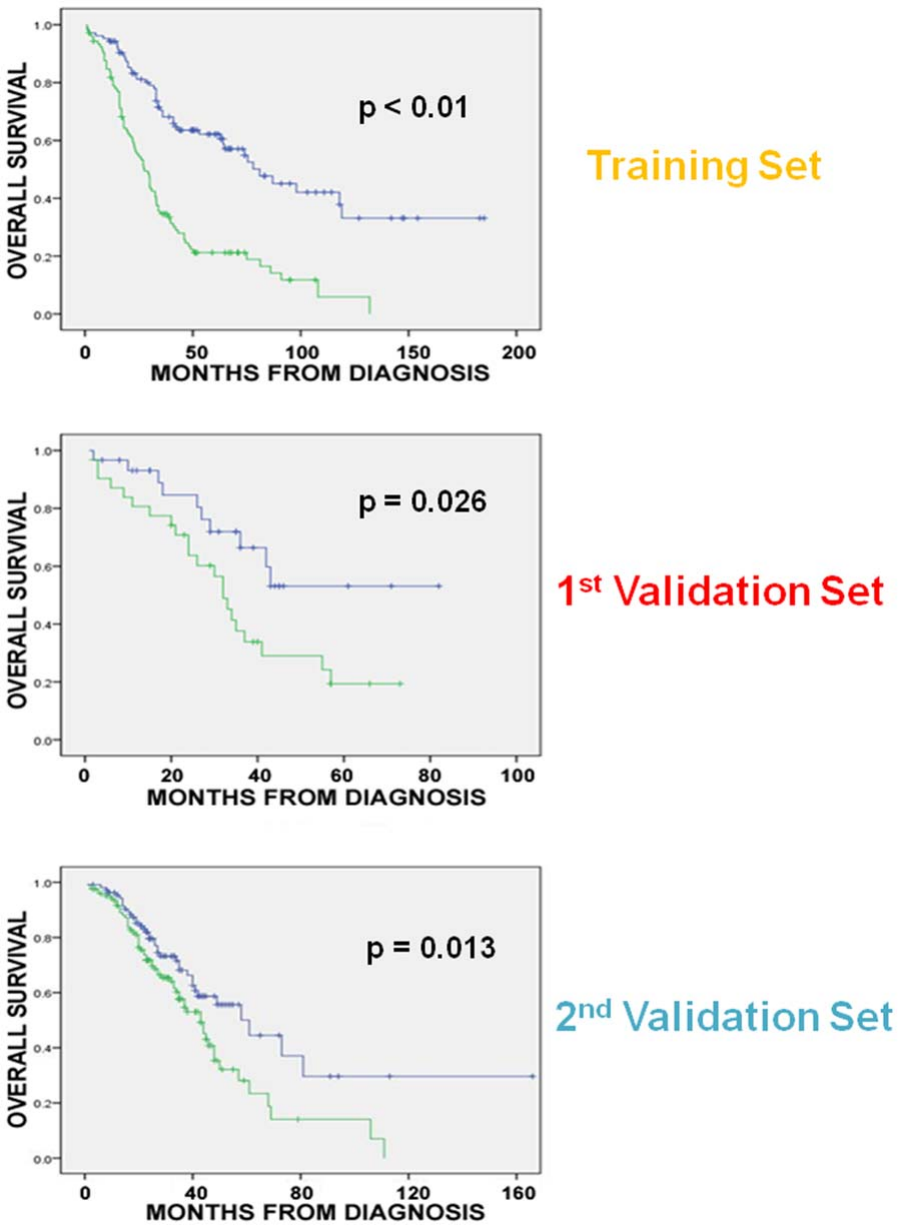
Association between 19-gene model and overall survival in the training and validation sets. The 19-gene model distinguished between a high and a low-risk group in the training set with a median OS of 31 months and 78 months respectively (log rank p<0.01, permutation p = 0.02), a high and a low-risk group for OS in the 1^st^ validation set (median OS 32 months versus not-yet-reached respectively, log rank p = 0.026), and a high and a low-risk group for OS in the 2nd validation set (median OS 43 months versus 61 months respectively, log rank p = 0.013).

Since our goal was to develop oligogene prognostic signatures we first identified models with the lowest number of genes that could provide prognostic information in the integrated training set. Models with as few as 2 genes distinguished between a high and a low-risk group for survival in the combined training set (HR = 1.7, p = 0.003). Then, we evaluated models with higher number of genes in the training set and noticed progressively increased hazard ratios (HRs) until there was a plateau, with stable, statistically significant HRs between 14 and 19 genes (i.e. HR = 2.1–2.3, p<0.001). Of these models, the 19-gene model showed the best prognostic performance as evident by its higher hazard ratio compared to the others. The best prognostic model (19 genes, Table 2) distinguished between a high and a low-risk group (median OS 31 and 78 months respectively, log rank p<0.01, permutation p = 0.02) (Figure 3).

**Table 2:** Genes and Probe Sets That Constitute the 19-Gene Model.

GENE SYMBOL	PROBESET	GENE NAME
INVS	210114_at	Inversin
CACNG1	206612_at	calcium channel, voltage-dependent, gamma subunit 1
ANXA2P1	210876_at	annexin A2 pseudogene 1
NEBL	217585_at	Nebulette
PAGE1	206897_at	P antigen family, member 1 (prostate associated)
MPZL2	203780_at	myelin protein zero-like 2
MAP3K10	206362_x_at	mitogen-activated protein kinase kinase kinase 10
H6PD	206933_s_at	hexose-6-phosphate dehydrogenase (glucose 1-dehydrogenase)
ANXA2P3	211241_at	annexin A2 pseudogene 3
CAMP	210244_at	cathelicidin antimicrobial peptide
PLD2	209643_s_at	phospholipase D2
CTSZ	212562_s_at	cathepsin Z
SLC12A4	209401_s_at	solute carrier family 12 (potassium/chloride transporters), member 4
RBP3	210318_at	retinol binding protein 3, interstitial
TPO	210342_s_at	thyroid peroxidase
LBX1	208380_at	ladybird homeobox 1
ASGR1	206743_s_at	asialoglycoprotein receptor 1
DMN	214304_x_at	Desmuslin
KRT31	206677_at	keratin 31

### Independent validation of the multi-gene prognostic classifiers

The 19-gene prognostic classifier was applied without any further modification to the 1^st^ validation set which included expression data obtained from an independent cohort of advanced stage ovarian cancers (Table 1, n = 61) using our custom array containing the 650 previously selected genes; these genes had been selected without prior knowledge about their prognostic performance in the validation set. The 19-gene model distinguished between a high and a low-risk group (median OS 32 months versus not-yet-reached respectively, log rank p = 0.026, at 33 months median follow up, Figure 3). Of note, when we prioritized the 19 genes based on their correlation with the principal components of the dataset or the weight of their contribution to the model, classifiers including the top 8–19 genes were also prognostically valid in the 1st validation set (Text S1).

The 19-gene prognostic classifier was also applied without any further modification to the 2nd validation set which included expression data from 229 ovarian cancers (Table 1, n = 229). Again, the 19-gene model distinguished between a high and a low-risk group (median OS 43 months versus 61 months respectively, log rank p = 0.013, Figure 3). Similar to the 1^st^ validation set, when we prioritized the 19 genes based on their correlation with the principal components or their weight of contribution to the model, several classifiers including the top 8–19 genes were also prognostically valid in the 2nd validation set (Text S1).

Importantly, we tried to reproduce the prognostic power of two previously reported signatures, from the BIDMC and DUKE datasets, respectively [6], [10]. Neither signature was reproducible in either of the two independent validation sets (Text S1). Reasoning that this may be due to different analytical algorithms applied in the previous studies, we attempted to build new signatures using the supervised principal component survival method separately in each of the 4 datasets that comprised the integrated training set. Again, none of these signatures could be validated in either of the two independent sets (Text S1). These observations underscore the value of integrating multiple expression datasets in order to derive widely reproducible signatures.

### Independent prognostic significance of the classifier adjusted for known clinical and pathologic prognostic factors

**Figure 4:**
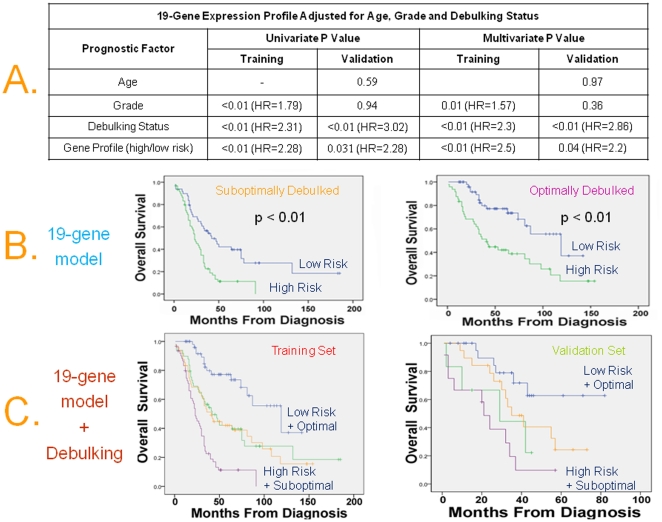
Independent prognostic significance of the multigene classifiers adjusted for known clinical and pathologic prognostic factors. **A**) Prognostic value of the 19-Gene expression profile adjusted for known prognostic factors by Cox Proportional Hazards Regression in the training and 1^st^ validation sets. **B**) Kaplan-Meier analysis for OS as a function of the 19-gene profile for homogeneous subsets of patients with optimal and suboptimal debulking status in the training set. **C**) The combination of optimal debulking and low-risk 19-gene profile was associated with a median OS of 119 months in the training set and not-yet-reached in the validation set, while the combination of suboptimal debulking and high-risk 19-gene profile was associated with a median OS of 23 months in the training set (HR = 7.3, 95% C.I. 3.4–13.5) and 21 months in the 1^st^ validation set (HR = 5.8, 95% C.I. 2.1–16).

We performed multivariate analysis and formally established that the 19-gene model maintained independent prognostic significance adjusted for confounding factors, in both training and the two independent validation sets (Figure 4A and Table 3). Specifically, the Hazard Ratio (HR) of death for the unfavorable versus the favorable group was 2.47 in the training set (95% CI, 1.71 to 3.56; p<0.01), 2.2 in the 1^st^ validation set (95% CI, 1.01 to 7.76; p = 0.04), (Figure 4A) and 1.59 in the 2^nd^ validation set (95% CI, 1.05 to 2.4; p = 0.03), (Table 3). Because only 8/229 (3%) of the tumors were definitely known to be suboptimally debulked in the 2^nd^ validation set, debulking status was included in the multivariate analysis of the 2^nd^ validation set as “grossly visible” versus “no visible” residual disease after surgery. Notably the independent prognostic value of the profile held true regardless of whether low grade was defined as grade 1 or grade 1 and 2 disease (Table 3).

**Table 3:** Multivariate Analysis in the 2^nd^ Validation Set.

19-Gene Profile Adjusted for Age, Grade, Stage and Residual Disease* in 2^nd^ Validation Set		
Prognostic Factor	Univariate P Value	Multivariate P Value
Age	**0.035 (HR = 1.56)**	0.06
Grade^a^	0.452	0.96
Stage	0.267	0.205
Grossly Visible vsNo Visible	**0.003 (HR = 2.32)**	**0.008 (HR = 2.21)**
Gene Profile (high/low risk)	**0.015 (HR = 1.65)**	**0.03 (HR = 1.59)**

a Grade 1 tumors were removed from this dataset (Table 1). If we also remove grade 2 tumors from the 2nd validation dataset (as grade 2 disease can also be considered low grade), the 19-gene profile was again associated with overall survival both in univariate (HR = 1.8, 95% C.I. 1.09–2.99) and multivariate analysis (HR = 1.91, 95% C.I. 1.13–3.26).

Data on chemotherapy response were available only for the 1st validation set. When we included chemotherapy response (i.e. achievement of complete clinical response (CCR) after first line chemotherapy versus no achievement of CCR) in the multivariate analysis for the 1st validation set, the 19-gene profile maintained its independent prognostic significance (HR = 3.96, 95% C.I. 1.56–10.1;p = 0.004).

Figure 4B also shows that the 19-gene profile was still prognostic of OS when applied in the homogeneous subsets of patients with optimal and suboptimal debulking status in the training set. This subset analysis could not be performed in the 1^st^ validation set because of sample size limitations, and in the 2^nd^ validation set because only 8/229 tumors (3%), were definitely known to be suboptimally debulked.

Gene expression models and debulking status were the strongest independent predictors of survival; therefore we were interested to assess their combined prognostic power, which is also shown in Figure 4C. Notably, the combination of optimal debulking and low-risk 19-gene profile was associated with a median OS of 119 months in the training set and not-yet-reached in the 1^st^ validation set, while the combination of suboptimal debulking and high-risk 19-gene profile was associated with a median OS of 23 months in the training set (HR = 7.3, 95% C.I. 3.4–13.5) and 21 months in the 1^st^ validation set (HR = 5.8, 95% C.I. 2.1–16) demonstrating that the combination of the two variables is much more powerful than either of them individually. This combination could not be assessed in the 2^nd^ validation set because only 3% of the tumors were definitely known to be suboptimally debulked.

### Genome-wide molecular correspondence of high and low-risk groups between the training and validation sets

**Figure 5:**
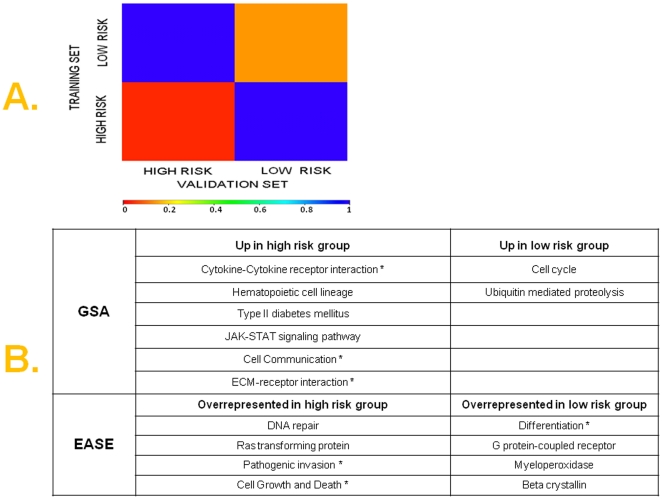
A) Genome-wide molecular correspondence of high and low-risk groups between training and 1^st^ validation set. SubMap analysis of genome-wide correspondence (similarity) between respective high and low risks groups in the training and 1^st^ validation set. The legend shows the relationship between color and FDR-adjusted p-values. Red color denotes high confidence for correspondence; blue color denotes lack of correspondence (Table S1). **B) Functional gene set analysis and functional representational analysis in high and low-risk disease samples.** Gene set analysis (GSA) over a wide range of differentially expressed genes revealed 8 pathways that were consistently statistically significantly differentially expressed. (Efron-Tibshirani GSA, p<0.05). Selected pathways-gene sets are shown that were overrepresented among high-risk and low-risk tumors by functional representational analysis using EASE (within-system FDR ≤0.01). A full list of these pathways is found in Tables S2, S3 and S4. Asterisks (*) denote pathways that were similarly expressed in corresponding prognostic groups in the 2^nd^ validation set.

It is frequently unclear if prognostic gene expression models are surrogates for underlying wider molecular or biological phenotypes, or simply a combination of individual prognostic genes. In order to test the hypothesis that our prognostic models are tracking molecular phenotypes of high versus low risk ovarian cancer, we used a methodology (Subclass Mapping-SubMap) that is uniquely suitable to assess the genome-wide molecular correspondence of pre-specified subtypes in independent and even technically disparate datasets [17]. Specifically, we investigated whether high or low-risk tumors in the combined training set were molecularly homologous with high or low-risk tumors in the 1^st^ validation set, above and beyond the handful of genes contained in the models. This is done by demonstrating enrichment of the gene profile of the “high risk” (or “low risk”) group in the training set for a large number of markers genes for the “high risk” (or “low risk”) group in the validation set and vice versa. As shown in Figure 5A, for the 19-gene model, high and low-risk tumors in the combined training set corresponded with high degree of statistical certainty with high and low-risk tumors respectively in the validation set (Table S1). This result was reproduced using various subsets of marker genes for the 19-gene model.

For the 2^nd^ validation dataset, favorable (C3 and C6) and unfavorable (C1, C2, C4, C5) prognostic molecular subtypes had already been defined by the authors [15]. We therefore assessed whether these previously defined molecular subtypes were reproduced in the low and high-risk groups as defined by our 19-gene profile in the 2^nd^ validation set (Figure 3). Indeed, in the 2^nd^ validation set, the low risk group (as defined by the 19-gene profile) was enriched for the favorable (C3 and C6) subtypes and the high-risk group was enriched for the unfavorable subtypes, as previously defined [15] (2-sided Fisher's exact p = 0.0016).

### Pathway analysis in high and low-risk disease groups

In order to gain insight into the pathway complexity of high and low-risk disease, we performed pathway and representational analyses to identify annotated pathways and functional gene groups that were overrepresented (enriched) in the gene profiles of the two risk categories in the large training set (the custom array, by design, contained too few genes to perform this analysis in the validation set).

GSA pathway analysis was performed over a wide range of differentially expressed genes between high and low-risk groups [using a t-test p from 0.01 (3264 genes) to as low as 0.0001 (1698 genes)], and revealed eight pathways (Figure 5B) that were consistently statistically significantly differentially expressed (Efron-Tibshirani GSA test p <0.05).

We also performed functional representational analysis using EASE among genes that were upregulated and downregulated in the high- versus low-risk patients (using a t-test p<10^−6^). We found 22 and 54 pathways overrepresented among genes upregulated and downregulated in high-risk tumors respectively at a within-system FDR threshold of 0.01. A full list of these pathways is found in Tables S2, S3 and S4, while selected pathways are presented in Figure 5B.

Interestingly, several of these pathways (Figure 5B), that were upregulated in high risk tumors i.e. “cytokine-cytokine receptor interaction”, “cell communication”, “ECM-receptor interaction”, “pathogenic invasion”, “cell growth”, and low risk tumors i.e. “differentiation”, were also similarly expressed in the high and low-risk tumors as previously reported in the 2^nd^ validation set [15].

### Prognostic gene expression models reflect activation of known oncogenic pathways in individual tumor samples

Given that GSA or EASE cannot assign pathway activation status for individual tumor samples, we applied previously developed gene expression “readouts” resulting from experimentally controlled activation of specific oncogenic pathways (src, b-catenin and E2F3) that have been shown to bear prognostic relevance in ovarian cancer [12]. We discovered that in the 239-tumor training set, the odds-ratios of activation of src and b-catenin pathways in our high versus low-risk group were 3.42 (95% C.I. 1.89–6.18) and 2.77 (95% C.I. 1.59–4.8) respectively, while the odds-ratio for E2F3 was 0.251 (95% C.I. 0.141 – 0.446). This is consistent with previous studies indicating that activation of src and b-catenin pathways are associated with poor outcome while activation of E2F3 is associated with good outcome, and indicates that our analysis captures biologically relevant information that is not immediately obvious by examining the content of the 19-gene profile. In multivariate analysis including the 19-gene model and the 3 oncogenic pathways, the 19-gene model maintained independent prognostic significance, whereas the activation patterns of the oncogenic pathways did not (data not shown).

## Discussion

Although the suitability of gene expression profiling for prognostication has been demonstrated in ovarian cancer [6], [8], [10], several challenges must be addressed before it becomes a clinically useful tool. Previous prognostic microarray studies were limited by sample size, interlaboratory variability, lack of external (out of study) validation, non-standardized analytical approaches and inclusion of histologic subtypes with distinct genetic profiles and outcome (i.e. clear cell, and mucinous cancers) [11]. In this study we described a successful pipeline that may also be useful for similar efforts in other cancers. We reprocessed and integrated raw data from four separate, previously generated microarray datasets [10], [12], [13], [14] originating from different laboratories and run on different platforms, into a large and homogeneous set, excluding mucinous and clear cell EOCs, thus maximizing our power to identify robust profiles while minimizing false positive findings. We corrected the non-biological experimental variation (“batch effect”) [18], which was clearly evident across studies (Figure 2) and composed a final training cohort of 239 tumors. We also used a standardized survival analysis method that compares favorably to other methods applied on microarray data [16], [19]. The resulting prognostic model was validated twice, in two separate independent sets. This is the first time, to our knowledge, that this has been attempted in this disease. Tumors included in the two validation cohorts originated from different institutions and were run in different laboratories and time periods than the tumors included in the combined training cohort. A custom chip was used for the 1^st^ validation set, and a large publicly available whole-genome dataset was used as a 2^nd^ validation set, while the training samples were previously run on different (whole-genome) platforms many years earlier. In addition to the rigor of this validation process, our use of publicly available datasets and of a customized design chip minimizes the cost of introducing gene-profiling technology to routine clinical practice.

A 19-gene model with optimal prognostic performance in the training set discriminated between a high and a low-risk group for OS in the two validation sets, while maintaining its independent association with survival in multivariate analysis adjusting for known clinicopathologic confounding factors. Of note, previously reported gene expression signatures from individual component datasets of the training set [6], [10], or newly generated models using our current methodology in these datasets, were not reproducible in either of the two independent validation datasets. This suggests that our strategy of integrating information from different and technically disparate datasets into a composite training set augments our ability to capture widely reproducible prognostic gene expression patterns. The variability in Hazard Ratio estimates for the 19-gene profile between the training and two validation sets, likely reflects the differences between the various clinical cohorts, whose characteristics are rarely identical in microarray studies. For instance, the 2^nd^ validation set appears to overwhelmingly consist of optimally debulked, thus improved prognosis patients. Nonetheless, this further underscores the validity of the profile when applied to a wide range of ovarian cancer patient populations.

Gene expression models were as powerful as debulking status, the strongest known clinical predictor of survival in advanced EOC [4], and the combination of optimal debulking and low-risk profile defined a population with a long survival plateau (70% 5-year survival in both training and 1^st^ validation set). Conversely, the combination of suboptimal debulking and high-risk profile defined a population with only 10% 5-year survival. Such powerful prognostic stratification in advanced EOC is not possible using conventional clinical factors at the time of diagnosis and may be useful for stratification of high-risk patients that are considered for investigational approaches using maintenance and/or consolidation strategies, or low-risk medically unstable patients that may avoid the relatively toxic intraperitoneal chemotherapy [20].

Our study also aimed to investigate wither the profile is not simply a mathematical combination of 19 prognostic variables, but is also tracking molecular phenotypes of high- versus low-risk ovarian cancer. Using a methodology (SubMap) that is uniquely suitable to assess the wider genomic resemblance of subtypes identified in multiple, independent, and disparate datasets [17], we confirmed that the low and high-risk groups assigned by our prognostic models were molecularly homologous between training and validation sets, suggesting that we have not simply validated a mathematical prognostic function but also true molecular phenotypes of good- and poor-outcome. In the 2nd validation set, molecular outcome subtypes had already been established by the authors [15]. Our finding that these molecular subtypes were overrepresented (enriched) in the high and low risk groups identified by our 19-gene profile, further attests to the notion that the profile is tracking true and reproducible outcome phenotypes in EOC.

While it was beyond the scope of our study to investigate the precise biological role of any specific pathway, it is noteworthy that pathways that were upregulated in the high- risk group have been implicated in ovarian carcinogenesis and/or associated with aggressive disease and poor outcome [21], [22], [23]. Furthermore, pathways that were overrepresented among genes overexpressed in high-risk tumors have been also been associated with inferior outcome [24], [25], [26], lending biologic plausibility to the phenotypes we discovered. Importantly, several of these pathways (Figure 5B) were also similarly expressed in the high and low-risk tumors previously reported in the publicly available 2^nd^ validation set, demonstrating reproducibility of biological networks associated with good and bad-outcome between the different datasets [15].

Finally, we took advantage of previously developed gene expression “read outs” resulting from experimentally controlled oncogenic pathway activation (src, b-catenin and E2F3) to assess activation status in individual tumor samples [12], [27]. Although there is an ongoing debate about how the oncogenic pathway analysis described by Bild et al. [12]. was applied in one particular study [28], the original oncogenic pathway analysis method described by Bild et al. has not been challenged. Consistent with known prior data, src and b-catenin pathways were more frequently activated in high-risk compared to low-risk tumors, while the opposite was true for the E2F3 pathway [12], [27], [29]. The novel association of the oncogenic pathway activation status with a phenotype “captured” by a marker 19-gene profile, of which none of the pathway genes is a member, demonstrates that biological inference in microarray studies should not be limited to the frequently applied approach of screening a list of top marker genes in a prognostic signature. Of note, these oncogenic pathways lost independent prognostic significance in multivariate analysis when the profile was included, suggesting that our prognostic classifier is capturing complex phenotypes and that outcome differences in ovarian cancer may not be adequately explained by deregulation of a single oncogenic or signaling pathway.

In conclusion, our approach exemplifies how integration and disciplined analysis of the rich information content of published but disparate cancer microarray datasets can overcome previous limitations and lead to development of robust and potentially widely applicable prognostic classifiers. A custom array may also be a practical tool in the study and management of cancer. Our study is consistent with, but provides complementary insight to previous seminal work that demonstrated the reproducibility of various gene expression profiles, when multiple array cohorts were run simultaneously, using the same protocol, on the same microarray platform in different laboratories [30].

The success of our approach does not negate the importance of previous individual expression studies, which have identified gene patterns with clinical and biologic relevance; rather effective integration of these studies may represent an important step forward towards wider clinical application of gene expression assays.

## Materials and Methods

### Synthesis of the combined training set

Raw gene expression profile data (Affymetrix .CEL files) were retrieved from four previously reported clinically annotated microarray datasets from different institutions (Beth Israel Deaconess Medical Center (BIDMC) [10], University of Pennsylvania (PENN) [14], Duke (http://data.genome.duke.edu/oncogene.php) and MD Anderson (http://www.mdanderson.org/departments/expther/bastovcalab/) comprising 265 advanced stage (stages 3 and 4) papillary serous ovarian cancers. These were profiled on Affymetrix U95Av2 (BIDMC and MD Anderson), Affymetrix U133A (Duke) and Affymetrix U133 Plus 2.0 (Penn) array platforms (the hybridization protocols are commercially available, websites in Text S1).

These were reprocessed uniformly and combined into a single training dataset: First, signal intensity was normalized within each individual dataset using Robust Multi-Array Average (RMA) analysis. Probesets were mapped across different platforms using Affymetrix annotation files and the Affymetrix ‘best match’ tool. In order to minimize the possibility that there are experimental batch effects that associate with OS within each individual dataset, we performed unsupervised hierarchical clustering in each of the 4 datasets. In all cases, we observed 2 predominant clusters, and there was no statistically significant difference in overall survival between these 2 predominant clusters in any of the datasets. Coinertia analysis [31] was performed in order to determine the loss of information incurred by reducing the number of genes to the subset common to the different Affymetrix platform. The overall similarity between datasets in the set of common samples was measured using a multivariate extension of the Pearson correlation coefficient called the RV-coefficient. The RV-coefficient is calculated as the total co-inertia (sum of eigenvalues of a co-inertia analysis) divided by the square root of the product of the squared total inertias (sum of the eigenvalues) from the individual COAs. It has a range 0 to 1 where a high RV-coefficient indicates a high degree of co-structure. When the subset of common genes was examined, the RV co-efficient was 0.86 indicating that the global correlation structure between datasets was not affected by the reduction in genes. Hence we decided to just examine the common genes in subsequent analysis.

Following normalization, technical outlier samples were identified and excluded using a model-based quality control assessment that included relative log2 expression (RLE) measures, normalized un-scaled standard errors (NUSE), array pseudo-images, and the percent of probe sets called “present”. Finally, systemic non-biological interlaboratory experimental variation (“batch effect”) between the datasets was adjusted using non-parametric empirical Bayes frameworks implemented in ComBat (http://statistics.byu.edu/johnson/ComBat) [18]. The resulting normalized, standardized dataset contained 239 samples.

### Independent Validation Sets

The 1^st^ independent validation set included 61 patients with advanced stage (III, IV) ovarian cancer diagnosed between 9/2001 and 7/2008 at BIDMC, Massachusetts General, and Brigham and Women's hospitals (Table 1). All patients underwent standard surgical and chemotherapy treatment [1]. The study protocol for collection and use of tissue and clinical information for all patients was approved by the institutional review boards at Beth Israel Deaconess Medical Center, Massachusetts General Hospital, Brigham and Women's Hospital and Dana Farber Cancer Institute. All patients provided written informed consent authorizing the collection and use of the tissue for study purposes. Profiling was performed using a custom Affymetrix GeneChip™ with 650 genes. After we had integrated the 4 aforementioned datasets into one combined training set, and developed and validated the prognostic models in the 1^st^ independent validation set using the custom Affymetrix GeneChip^TM^, another ovarian cancer dataset became publicly available and thus we decided to use it as a 2^nd^ independent validation set (GSE9899) [15]. This dataset included 229 patients with epithelial ovarian, primary peritoneal, or fallopian tube cancer (after excluding patients with grade 1 disease, and those with no survival data), diagnosed between 1992 and 2006. Profiling was performed using Affymetrix U133 Plus 2.0 arrays. The clinicopathologic characteristics for both validation sets are shown in Table 1.

### Design of Custom Array Chip

Gene-expression profiling in the 1st independent validation dataset, was performed using a custom Affymetrix GeneChip (data available at GEO, accession number GSE19161, http://www.ncbi.nlm.nih.gov/geo/query/acc.cgi?token=bdgzfwmysouamxe&acc=GSE19161). The number of probe sets on the custom chip represents a compromise between cost and inclusion of an adequate pool of top candidate prognostic genes, *a priori* identified by applying the supervised principal component survival analysis method in each of the individual training datasets. Specifically, we applied supervised principal components analysis [16] in each dataset and selected subsets of probe sets that created a maximum significant survival split. Then, we applied the models to the remaining training datasets and identified the maximum number of probe sets that could also generate a significant survival splits in all remaining training datasets. This analysis was repeated for the different training datasets, ultimately generating a pool of markers that was the union of probe sets resulting from these analyses. This pool, including probe sets that were part of significant prognostic models in all datasets, was selected for the custom array. We also added pre-defined prognostic signatures from previous publications [6], [10]. Next, we screened the list for duplicates and if there were duplicates we retained only the U 133-based probe set. Finally, we added a set of control spikes and normalization probe sets. The total number of probe sets was set to approximately 1200 (the precise number was 1191), based on the maximum number of probe sets that could be custom-spotted at the minimal possible cost. The array contained a total of 658 unique prognostic genes (excluding controls, averaging probe sets that were duplicates of the same gene, and converting all probe sets to the U 133 A platform). The design was carried out in collaboration with the Affymetrix technical design team.

### Development of prognostic models using the supervised principal component survival algorithm

Firstly, genes associated with survival in the combined training set at a significance level of 0.05 (Cox proportional hazards model) were identified. More details are included in Text S1. These genes were subsequently ranked based on their absolute Cox regression coefficients, and prognostic models with several sets of top ranking genes using the supervised principal component survival algorithm were developed. The methodological principles of the Survival Risk Prediction Algorithm have been previously described [16].

A high and a low-risk survival groups were defined based on a multivariate model of the expression level of the genes contained in each set of top ranking genes and the Cox regression coefficient for each gene. This multivariate model was used in a leave-one-out cross validation process to assign risk-group membership for clinical samples. Kaplan-Meier OS curves were plotted for two risk groups, with higher or lower than median risk of death or recurrence. Statistical significance of the survival splits was assessed by the log-rank test and a permutation statistic was calculated by randomly reassigning the survival data among cases, repeating the entire survival risk prediction 100 times and estimating how many times the log-rank statistic is lower than the log-rank statistic for the real data. This represented the permutation significance level for testing the null hypothesis that there is no relation between the expression data and survival.

These models were directly applied without any modification to the two validation sets after adjustment of non-biological experimental variation across data sets [18]. Multidimensional plots of the training, validation 1 and validation 2 datasets, before and after correction of batch effect show that before application of the batch adjustment algorithm, each dataset clearly separated from all the others (“batch effect”), whereas after correction of batch effect, samples from all datasets were well intermixed (Figure S1). All reported p-values are two-sided. Multivariate adjustment for known prognostic factors was performed using a Cox proportional hazards regression model that included grade, age (<65 years versus ≥65 years), stage (early stage 1 or 2 versus advanced stage 3 or 4), debulking status (optimal, less than or equal to 1 cm.; or suboptimal, greater than 1 cm. residual disease) and 19-gene profile risk status (low- vs high-risk). Because only 8/229 (3%) of the tumors were definitely known to be suboptimally debulked (based on the 1 cm cutoff criterion for debulking status) in the 2^nd^ validation set, debulking status was included in the multivariate analysis of the 2^nd^ validation set as “grossly visible” versus “no visible” residual disease after surgery.

### Genome-wide molecular correspondence of high and low-risk groups between the training and validation sets using Subclass Mapping (SubMap)

In order to assess whether tumors assigned as high or low-risk in the combined training set were molecularly homologous with tumors assigned as high or low-risk, respectively, in the validation set, we used a recently developed methodology (Subclass Mapping-SubMap) that is uniquely suitable to assess the genome-wide molecular correspondence of pre-specified subtypes in independent and even technically disparate datasets.

Submap is an unsupervised subclass mapping method that identifies the correspondence or commonality of subtypes found in multiple, independent data sets potentially generated on different platforms. Suppose we have two independent datasets A and B with i and j candidate subclasses respectively (a subclass must contain at least 10% of the samples of a dataset to be considered as a candidate subclass). Marker gene lists ‘marker(Ai)’ for each candidate subclass in A (A1, …Ai) are determined based on the differential gene expression versus the rest of A subclasses, while genes in data set B are rank-ordered according to their correlation with each Bj subclass versus the other B subclasses to yield a gene list, ‘ranking(Bj)’. Association between Ai and Bj is evaluated by quantifying the over-representation of ‘marker(Ai)’ in the up-regulated end of the list ‘ranking(Bj)’ using Gene Set Enrichment Analysis (GSEA) as previously described [32]. An enrichment score (ESAiBj) is calculated, and statistical significance is assessed as a p-value, pAiBj, by randomly permuting sample class labels in B and estimating the null distribution of ESAiBj score. This process is repeated by interchanging the role of A and B to compute ESBjAi and pBjAi. Mutual enrichment information is defined by combining pAiBj and pBjAi using the Fisher inverse chi-square statistic, Fij. Statistical significance is estimated based on a null distribution for the Fij generated by randomly picking the p from corresponding null distributions for ESAiBj and ESBjAi. A FDR adjustment to account for multiple hypotheses testing is performed, and FDR adjusted p-values are summarized in the subclass association matrix (SA matrix).

### Pathway Analysis

In order to assess whether the gene expression profiles of high- and low-risk disease samples were enriched for specific functional groups of genes, we performed functional category representational analysis using EASE [33] among genes that were upregulated and downregulated in the high- versus low-risk patients (using a t-test p<10^−6^). We analyzed representation of Gene Ontology assignments, phenotype, PFAM, PIR, Swiss-Prot keywords, GenMAPP and KEGG pathways. For each functional category of genes we utilized a False Discovery Rate (FDR) of ≤0.01 to assess the impact of multiple testing.

Furthermore, we performed gene set analysis (GSA) as described by Efron and Tibshirani [34], which is an evolution of the previously reported Gene Set Enrichment Analysis (GSEA). GSA pathway analysis was performed over a wide range of differentially expressed genes between high and low-risk groups [using a t-test p from 0.01 (3264 genes) to as low as 0.0001 (1698 genes)], to identify pathways that were consistently statistically significantly differentially expressed at a GSA p<0.05.

### Prediction of probability of oncogenic pathway activation in tumor samples

We used signatures of experimentally controlled oncogenic pathway activation that are publicly available at http://dig.genome.duke.edu/. These signatures have been validated in a variety of in vitro models and patient samples. Specific mathematical models based on the Bayesian probit regression algorithm, estimating the probability of activation of each pathway were trained in the experimental systems used to develop these signatures (arrays available at the website indexed above) and applied on individual tumor samples of the training set. Non-biological experimental variation between the experimental system arrays and the ovarian cancer datasets was corrected using a previously described batch effect adjustment algorithm, ComBat (http://statistics.byu.edu/johnson/ComBat) [18]. Each individual tumor sample was assigned a probability value of pathway activation, from 0 to 1. A probability value higher than 0.5 was used as cut off for pathway activation. The association between gene expression profile risk status (i.e. low versus high-risk) and activation of oncogenic pathways in each tumor sample was tested using 2×2 table statistics and Odds-ratios.

## Supporting Information

Figure S1 **Adjustment for non-biological experimental variation.** Multidimensional scaling of the integrated training and validation sets revealed that, before application of the batch adjustment algorithm, each dataset clearly separated from all the others (“batch effect”), whereas after correction of batch effect, samples from all datasets were well intermixed.
(TIF)

Table S1 Assessment of genome-wide molecular correspondence of high and low-risk groups between the training and validation sets using SubMap
(DOC)

Table S2 Pathways identified by GSA and their Efron-Tibshirani p values
(DOC)

Table S3 Pathways overrepresented among genes upregulated in high-risk tumors by EASE
(DOC)

Table S4 Pathways overrepresented among genes upregulated in low-risk tumors by EASE
(DOC)

Text S1 Supplementary Text
(DOC)
