# Supplementary figures and images for: Integrated Analysis of Multiple Microarray Datasets Identifies a Reproducible Survival Predictor in Ovarian Cancer
Source: PLoS One. 2011 Mar 29;6(3):e18202. doi: 10.1371/journal.pone.0018202 (PMC3066217; doi:10.1371/journal.pone.0018202)

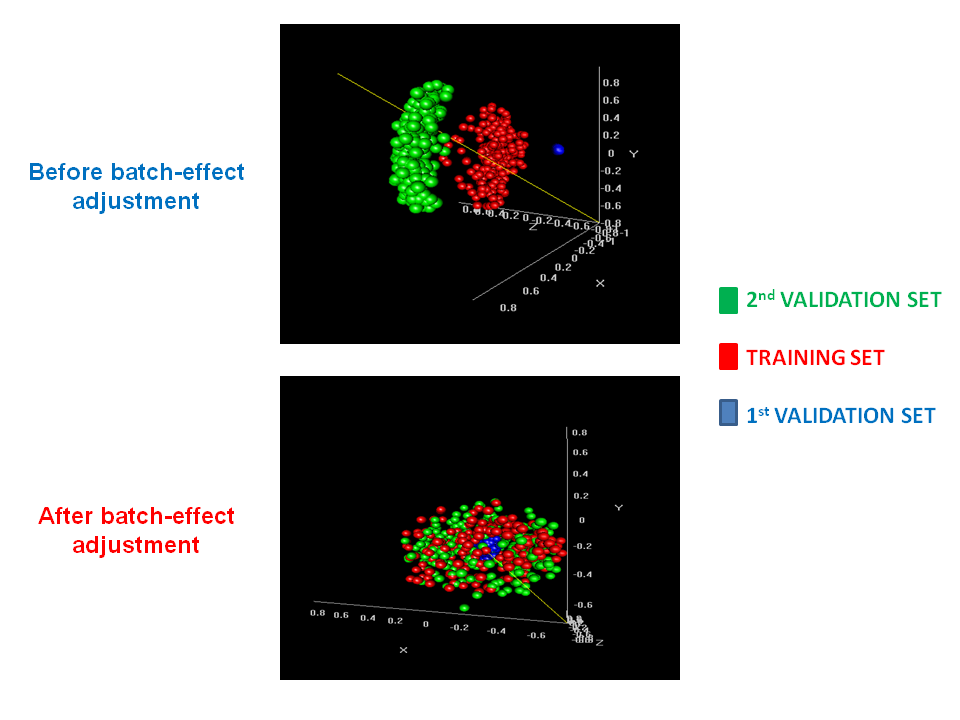

Supplement: Figure S1 — Adjustment for non-biological experimental variation. Multidimensional scaling of the integrated training and validation sets revealed that, before application of the batch adjustment algorithm, each dataset clearly separated from all the others (“batch effect”), whereas after correction of batch effect, samples from all datasets were well intermixed. (TIF) [file pone.0018202.s001.tif]
