# Supplementary material for: Integrated Analysis of Multiple Microarray Datasets Identifies a Reproducible Survival Predictor in Ovarian Cancer
Source: PLoS One. 2011 Mar 29;6(3):e18202. doi: 10.1371/journal.pone.0018202 (PMC3066217; doi:10.1371/journal.pone.0018202)
Supplement: Table S1 — Assessment of genome-wide molecular correspondence of high and low-risk groups between the training and validation sets using SubMap (DOC) [file pone.0018202.s002.doc]

| **Mutual enrichment permutation p value of Fisher inverse chi-square statistic** | | | | |
| --- | --- | --- | --- | --- |
|  | **Validation Dataset** | | | |
| **Training Dataset** | HIGH-RISK | | LOW-RISK | |
| LOW-RISK | 1 | | **0.07** | |
| HIGH-RISK | **0.001** | | 0.96 | |
| **Subclass Association Matrix (FDR Adjusted p-values)** | | | | |
|  | | **Validation Dataset** | | |
| **Training Dataset** | | HIGH-RISK | | LOW-RISK |
| LOW-RISK | | 1 | | **0.14** |
| HIGH-RISK | | **0.04** | | 1 |
