# Supplementary material for: Integrated Analysis of Multiple Microarray Datasets Identifies a Reproducible Survival Predictor in Ovarian Cancer
Source: PLoS One. 2011 Mar 29;6(3):e18202. doi: 10.1371/journal.pone.0018202 (PMC3066217; doi:10.1371/journal.pone.0018202)
Supplement: Table S2 — Pathways identified by GSA and their Efron-Tibshirani p values (DOC) [file pone.0018202.s003.doc]

| **GSA** | **Pathway** | **Efron test P value** |
| --- | --- | --- |
|  | Cytokine-Cytokine receptor interaction | 0.03 |
|  | Hematopoietic cell lineage | 0.03 |
|  | Type II diabetes mellitus | 0.03 |
|  | JAK-STAT signaling pathway | 0.04 |
|  | Cell Communication | 0.04 |
|  | ECM-receptor interaction | 0.04 |

|  | Cell cycle | 0.04 |
| --- | --- | --- |
|  | Ubiquitin mediated proteolysis | 0.04 |
