# Supplementary material for: Integrated Analysis of Multiple Microarray Datasets Identifies a Reproducible Survival Predictor in Ovarian Cancer
Source: PLoS One. 2011 Mar 29;6(3):e18202. doi: 10.1371/journal.pone.0018202 (PMC3066217; doi:10.1371/journal.pone.0018202)
Supplement: Table S3 — Pathways overrepresented among genes upregulated in high-risk tumors by EASE (DOC) [file pone.0018202.s004.doc]

| **Pathways** |
| --- |
| RNA splicing |
| Spliceosomal subunit |
| RNA-binding protein |
| Chromatin/chromosome structure |
| RNA-binding region RNP-1 (RNA recognition motif) |
| RNA processing/modification |
| [Zinc finger, C2H2 type, domain](http://us.expasy.org/cgi-bin/prosite-search-ac?PDOC00028) |
| AAA ATPase superfamily |
| Pathogenic Invasion |
| [Unassigned ribonucleoprotein repeat-containing proteins](http://pir.georgetown.edu/cgi-bin/ipcSF?id=SF015228) |
| [Protein export - Homo sapiens](http://www.genome.ad.jp/dbget-bin/show_pathway?hsa03060) |
| [Sorting and Degradation - Homo sapiens](http://www.genome.ad.jp/dbget-bin/show_pathway?hsa01230) |
| [Eukaryotic putative RNA-binding region RNP-1 signature](http://us.expasy.org/cgi-bin/prosite-search-ac?PDOC00030) |
| Prenylated cysteine |
| [Cell cycle - Homo sapiens](http://www.genome.ad.jp/dbget-bin/show_pathway?hsa04110) |
| [chromosome segregation protein SMC1](http://pir.georgetown.edu/cgi-bin/ipcSF?id=SF005251) |
| [pfam02259: FAT domain. The FAT domain is named after FRAP, ATM and TRRAP](http://pfam.wustl.edu/cgi-bin/getdesc?acc=PF02259) |
| Regulatory subunit |
| [Cell Growth and Death - Homo sapiens](http://www.genome.ad.jp/dbget-bin/show_pathway?hsa01420) |
| [Hs_Cell cycle](http://www.genmapp.org/MAPPSet-Human/KEGG_Pathway_MAPPs/Hs_Cell_Cycle.htm) |
| DNA repair |
| [ras transforming protein](http://pir.georgetown.edu/cgi-bin/pirwww/nbrfget?uid=FA2318&db=A) |
| RNA splicing |
