# Supplementary material for: Integrated Analysis of Multiple Microarray Datasets Identifies a Reproducible Survival Predictor in Ovarian Cancer
Source: PLoS One. 2011 Mar 29;6(3):e18202. doi: 10.1371/journal.pone.0018202 (PMC3066217; doi:10.1371/journal.pone.0018202)
Supplement: Table S4 — Pathways overrepresented among genes upregulated in low-risk tumors by EASE (DOC) [file pone.0018202.s005.doc]

| **Pathways** |
| --- |
| Channel [passive transporter] |
| ['Homeobox' domain signature](http://us.expasy.org/cgi-bin/prosite-search-ac?PDOC00027) |
| Pol II transcription |
| Homeobox domain |
| Chromoprotein |
| Homeobox |
| Chymotrypsin serine protease family (S1) |
| Glycoprotein |
| [Serine proteases, trypsin family, serine active site](http://us.expasy.org/cgi-bin/prosite-search-ac?PDOC00124) |
| Heme |
| Serine proteases, trypsin family |
| [Trypsin](http://pir.georgetown.edu/cgi-bin/ipcSF?id=SF001135) |
| Potassium channel |
| Photoreception |
| Serine proteinase |
| Cation channels (non-ligand gated) |
| Iron |
| Metalloprotein |
| [Human cytochrome P450 CYP2D6](http://pir.georgetown.edu/cgi-bin/pirwww/nbrfget?uid=FA1968&db=A) |
| [G-protein coupled receptors signature](http://us.expasy.org/cgi-bin/prosite-search-ac?PDOC00210) |
| [Vertebrate rhodopsin](http://pir.georgetown.edu/cgi-bin/ipcSF?id=SF002406) |
| Differentiation |
| G protein-coupled receptor |
| Receptor (signalling) |
| [Hs_Calcium Channels](http://www.genmapp.org/MAPPSet-Human/GenMAPP.org_MAPPs/Gene_Family_MAPPs/Hs_Calcium_Channels.htm) |
| [Serine proteases, trypsin family, histidine active site](http://us.expasy.org/cgi-bin/prosite-search-ac?PDOC00124) |
| [Human cytochrome P450 CYP2D6](http://pir.georgetown.edu/cgi-bin/ipcSF?id=SF000045) |
| [Hs_GPCRs, Class A Rhodopsin-like](http://www.genmapp.org/MAPPSet-Human/GPCRDB_MAPPs/Hs_GPCRs_Class_A_Rhodopsin-like.htm) |
| [Myeloperoxidase homology](http://pir.georgetown.edu/cgi-bin/pirwww/nbrfget?uid=DA4420&db=A) |
| [Blood group glycolipidbiosynthesis-lactoseries - Homo sapiens](http://www.genome.ad.jp/dbget-bin/show_pathway?hsa00601) |
| Rhodopsin-like GPCR superfamily |
| [Peroxidases proximal heme-ligand signature](http://us.expasy.org/cgi-bin/prosite-search-ac?PDOC00394) |
| CNS-specific functions |
| [Unassigned homeobox proteins](http://pir.georgetown.edu/cgi-bin/ipcSF?id=SF015223) |
| [Metabolism of Complex Lipids - Homo sapiens](http://www.genome.ad.jp/dbget-bin/show_pathway?hsa01180) |
| [Galactoside 3(4)-L-fucosyltransferase](http://pir.georgetown.edu/cgi-bin/ipcSF?id=SF005726) |
| [Beta-crystallin](http://pir.georgetown.edu/cgi-bin/ipcSF?id=SF002281) |
| [3'5'-cyclic nucleotide phosphodiesterases signature](http://us.expasy.org/cgi-bin/prosite-search-ac?PDOC00116) |
| [Gamma-Hexachlorocyclohexane degradation - Homo sapiens](http://www.genome.ad.jp/dbget-bin/show_pathway?hsa00361) |
| Other metabolism |
| [Hs_Peptide GPCRs](http://www.genmapp.org/MAPPSet-Human/GenMAPP.org_MAPPs/Gene_Family_MAPPs/Hs_Peptide_GPCRs.htm) |
| Retinitis pigmentosa, autosomal recessive |
| [G-protein coupled receptors family 3 signature 3](http://us.expasy.org/cgi-bin/prosite-search-ac?PDOC00754) |
| ['Paired box' domain signature](http://us.expasy.org/cgi-bin/prosite-search-ac?PDOC00034) |
| [G-protein coupled receptors family 3 signature 1](http://us.expasy.org/cgi-bin/prosite-search-ac?PDOC00754) |
| [Crystallins beta and gamma 'Greek key' motif signature](http://us.expasy.org/cgi-bin/prosite-search-ac?PDOC00197) |
| [G-protein coupled receptors family 3 signature 2](http://us.expasy.org/cgi-bin/prosite-search-ac?PDOC00754) |
| [Cytochrome P450 homology](http://pir.georgetown.edu/cgi-bin/pirwww/nbrfget?uid=DA4293&db=A) |
| [Homeobox domain](http://pfam.wustl.edu/cgi-bin/getdesc?acc=PF00046) |
| [Trypsin homology](http://pir.georgetown.edu/cgi-bin/pirwww/nbrfget?uid=DA1082&db=A) |
| [Myeloperoxidase](http://pir.georgetown.edu/cgi-bin/pirwww/nbrfget?uid=FA1978&db=A) |
| [Homeodomain](http://smart.embl-heidelberg.de/smart/do_annotation.pl?BLAST=DUMMY&ACC=SM00389) |
| [PDZ domain (Also known as DHR or GLGF). PDZ domains are found in diverse signaling proteins](http://pfam.wustl.edu/cgi-bin/getdesc?acc=PF00595) |
| [Domain present in PSD-95, Dlg, and ZO-1/2. Also called DHR (Dlg homologous region) or GLGF (relatively well conserved tetrapeptide in these domains). Some PDZs have been shown to bind C-terminal polypeptides](http://smart.embl-heidelberg.de/smart/do_annotation.pl?BLAST=DUMMY&ACC=SM00228) |
