# Supplementary material for: Integrated Analysis of Multiple Microarray Datasets Identifies a Reproducible Survival Predictor in Ovarian Cancer
Source: PLoS One. 2011 Mar 29;6(3):e18202. doi: 10.1371/journal.pone.0018202 (PMC3066217; doi:10.1371/journal.pone.0018202)
Supplement: Text S1 — Supplementary Text (DOC) [file pone.0018202.s006.doc]

**Supporting Methods**

**Selection of training and validation sets**

At the point that our project was initiated, there were only three publicly available clinically annotated ovarian cancer microarray datasets with survival data, i.e. Spentzos et al. (BIDMC - U95Av2 platform) [1], Bild et al. (DUKE - U133A platform) [2] and Marquez et al. (MD Anderson - U95Av2 platform) [3]. We also had access to another clinically annotated ovarian cancer Affymetrix U133 Plus 2.0 microarray dataset with survival data (Zhang et al. - PENN [4]) via personal communication. Only datasets with data run on Affymetrix U95 or later platform versions were included in our study, because earlier Affymetrix versions contained limited number of probesets, which would lead to a large loss of information when creating the final unified training dataset (the integrated training set contained only those probesets that were common to all Affymetrix platforms included in it).

Hybridization protocols for Affymetrix U95, U133A and U133 Plus 2.0 arrays are commercially available online at:

a.http://www.affymetrix.com/estore/browse/products.jsp?productId=131538&categoryId=35760&productName=GeneChip-Human-Genome-U95-Set#1_3

b.http://www.affymetrix.com/estore/browse/products.jsp?productId=131537&categoryId=35760&productName=GeneChip-Human-Genome-U133A-2.0-Array#1_3
c.http://www.affymetrix.com/estore/browse/products.jsp?productId=131455&categoryId=35760&productName=GeneChip-Human-Genome-U133-Plus-2.0-Array#1_3

In terms of all custom array Genechips designed by Affymetrix, protocols are available at: [http://media.affymetrix.com/support/downloads/manuals/expression_analysis_technical_manual.pdf](https://mail.caregroup.org/OWA/redir.aspx?C=fe48d8dc6c0b4b12a3a0100d422d1609&URL=http%3A%2F%2Fmedia.affymetrix.com%2Fsupport%2Fdownloads%2Fmanuals%2Fexpression_analysis_technical_manual.pdf)

We used these 4 datasets to construct the combined training set and design the custom array Affymetrix GeneChip by selecting the top candidate prognostic genes identified using the supervised principal component survival analysis method in each of the 4 datasets. The 19-gene model was validated in 2 independent validation sets. The first validation was performed using the custom array Affymetrix GeneChip in 61 patients from our institutions. For further validation of the 19-gene model, we used a second independent validation set (Tothill et al. [5]) that became publicly available after the integrated dataset was constructed and the custom array chip were designed. This dataset was selected based on two characteristics: a) it was run on Affymetrix U133 Plus 2.0 arrays and b) included patients from completely different institutions than those included in the 4 aforementioned datasets of the integrated training set.

**Development of multi-gene prognostic classifiers in the integrated training set**

As described in the materials and methods of the manuscript, we used the pool of the 650 marker genes in order to generate multi-gene prognostic classifiers in the integrated training set. Genes associated with survival (p < 0.05) were ranked based on their absolute Cox regression coefficients, and prognostic models with the top ranking genes were developed using supervised principal component survival analysis. Since our goal was to develop oligogene prognostic signatures we first identified models with the lowest number of genes that could provide prognostic information in the integrated training set. Models with as low as 2 genes distinguished between a high and a low-risk group for survival in the combined training set (HR=1.7, p=0.003). When we evaluated models with progressively more number of genes in the training set, we observed a plateau, with stable HRs (2.1-2.3, all statistically significant, p<0.001), between 14-20 genes (Table). Of these models, the 19-gene model exhibited the best prognostic performance as evident by its higher hazard ratio compared to the others. Importantly, in order to identify classifiers with even smaller number of genes, we selected further for the most informative genes by prioritizing the 19 genes based on their correlation with the principal components of the datasets and discovered a 8 gene model which showed similar prognostic performance as the 19-gene in the training set (HR=2.3, p<0.001). Performance of all these classifiers including the top 8-19 genes were prognostically valid in the validation sets as shown in the Supplement. The steps for development of the multigene classifiers are summarized in the following Table:

| **Steps for development of the multigene classifier** |
| --- |
| 1. Identify genes (from the 650 pool) associated with survival in the final combined training set at a significance level of 0.05 (Cox proportional hazards model) |
| 1. Rank these genes based on their absolute Cox regression coefficient |
| 1. Create prognostic models with several sets of top ranking genes using the supervised principal component survival algorithm in the combined training set |
| 1. Identify the lowest number of genes that gives statistically significant split in the training set (n=2, HR=1.7, p=0.003) |
| 1. Evaluate models with higher number of genes in the training set and progressively increased hazard ratios (HRs) until there is a plateau, with stable, statistically significant HRs (n=14-19 genes, HR=2.1-2.3, p<0.001) |
| 1. Further selection of the most informative genes by prioritizing the 19 genes based on their correlation with the principal components |

**Supporting Results**

**1. Performance of prognostic models with the top ranking genes based on their correlation with the principal components or the weight of their contribution to the 19-gene model in the 1^st^ validation set**

**18 GENES**

Median OS: 33 vs NYR months

Log Rank p = 0.068

HR: 1.98 (p = 0.074)

**16 GENES**

Median OS: 33 vs NYR months

Log Rank p = 0.068

HR: 1.98 (p = 0.074)

**14 GENES**

Median OS: 33 vs NYR months

Log Rank p = 0.028

HR: 2.38 (p = 0.034)

**12 GENES**

Median OS: 33 vs 43 months

Log Rank p = 0.13

HR: 1.76 (p = 0.14)

**10 GENES**

Median OS: 33 vs 43 months

Log Rank p = 0.1

HR: 1.87 (p = 0.11)

**8 GENES**

Median OS: 32 vs 55 months

Log Rank p = 0.033

HR: 2.28 (p = 0.038)

**2. Performance of prognostic models with the top ranking genes based on their correlation with the principal components or the weight of their contribution to the 19-gene model in the 2^nd^ validation set**


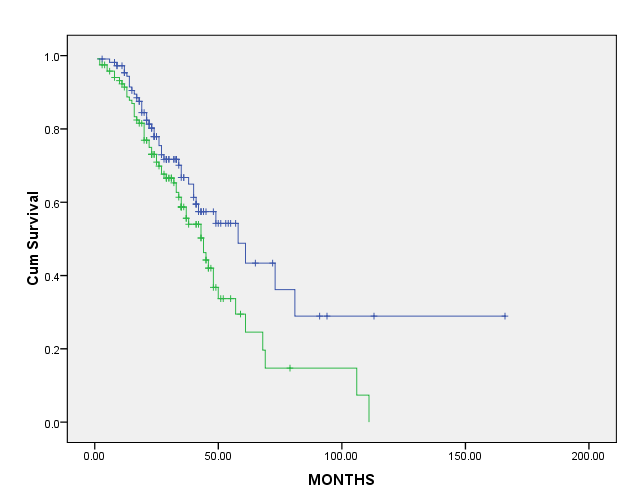


**18 GENES**

Median OS: 44 vs 58 months

Log Rank p = 0.034

HR: 1.53 (p = 0.037)


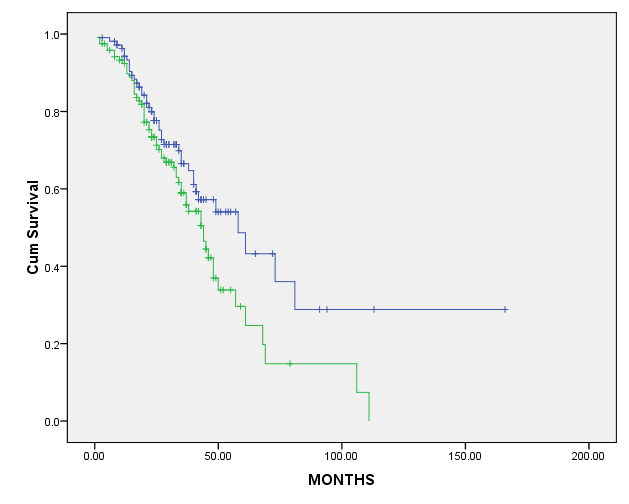


**16 GENES**

Median OS: 44 vs 58 months

Log Rank p = 0.045

HR: 1.5 (p = 0.048)


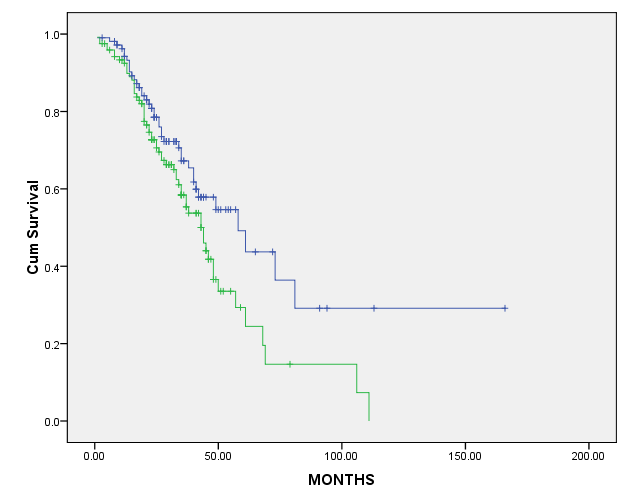


**14 GENES**

Median OS: 44 vs 58 months

Log Rank p = 0.031

HR: 1.55 (p = 0.033)


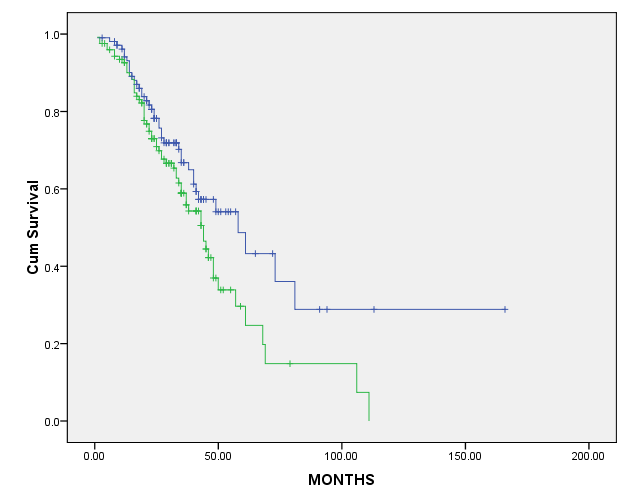


**12 GENES**

Median OS: 44 vs 58 months

Log Rank p = 0.042

HR: 1.51 (p = 0.045)


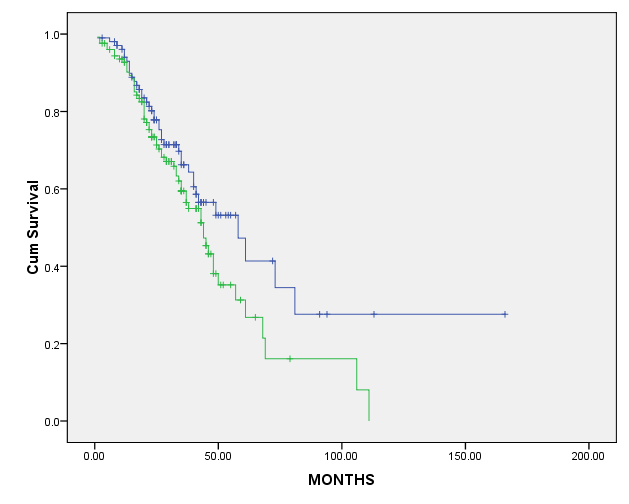


**10 GENES**

Median OS: 44 vs 58 months

Log Rank p = 0.077

HR: 1.43 (p = 0.081)


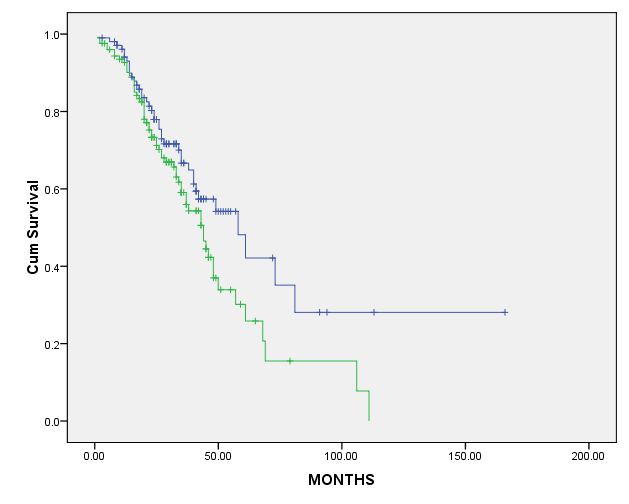


**8 GENES**

Median OS: 44 vs 58 months

Log Rank p = 0.053

HR: 1.49 (p = 0.056)

**3. Performance of previously reported prognostic models in 1^st^ validation set**

**BERCHUCK et al. Signature**

Log Rank p = 0.9

**SPENTZOS et al. Signature**

Log Rank p = 0.76

**4. Performance of prognostic models built using SPCA in each individual dataset in 1^st^ validation set**

**SPCA in BIDMC alone**

Log Rank p = 0.36

**SPCA in DUKE alone**

Log Rank p = 0.53

**SPCA in PENN alone**

Log Rank p = 0.3

**REFERENCES**

1. Spentzos D, Levine DA, Ramoni MF, Joseph M, Gu X, et al. (2004) Gene expression signature with independent prognostic significance in epithelial ovarian cancer. J Clin Oncol 22: 4700-4710.

2. Bild AH, Yao G, Chang JT, Wang Q, Potti A, et al. (2006) Oncogenic pathway signatures in human cancers as a guide to targeted therapies. Nature 439: 353-357.

3. Marquez RT, Baggerly KA, Patterson AP, Liu J, Broaddus R, et al. (2005) Patterns of gene expression in different histotypes of epithelial ovarian cancer correlate with those in normal fallopian tube, endometrium, and colon. Clin Cancer Res 11: 6116-6126.

4. Zhang L, Volinia S, Bonome T, Calin GA, Greshock J, et al. (2008) Genomic and epigenetic alterations deregulate microRNA expression in human epithelial ovarian cancer. Proc Natl Acad Sci U S A 105: 7004-7009.

5. Tothill RW, Tinker AV, George J, Brown R, Fox SB, et al. (2008) Novel molecular subtypes of serous and endometrioid ovarian cancer linked to clinical outcome. Clin Cancer Res 14: 5198-5208.
